# Immunomodulatory Molecules On Lung Cancer Stem Cells From Lymph Nodes Aspirates

**DOI:** 10.3390/cancers12040838

**Published:** 2020-03-31

**Authors:** Agata Raniszewska, Iwona Kwiecień, Rafał Sokołowski, Elżbieta Rutkowska, Joanna Domagała-Kulawik

**Affiliations:** 1 Department of Pathology, Medical University of Warsaw, Pawinskiego 7 Street, 02-106 Warsaw, Poland; 2 Department of Internal Medicine and Hematology Laboratory of Flow Cytometry, Military Medical Institute, Warsaw, ul. Szaserow 128, 04-141 Warsaw, Poland; ikwiecien@wim.mil.pl (I.K.); erutkowska@wim.mil.pl (E.R.); 3 Department of Internal Medicine, Pulmonology and Allergology, Military Medical Institute, Warsaw, ul. Szaserow 128, 04-141 Warsaw, Poland; rsokolowski@wim.mil.pl; 4 Department of Internal Medicine, Pulmonary Diseases and Allergy, Medical University of Warsaw, Banacha 1a Street, 02-097 Warsaw, Poland; domagalakulawik@gmail.com

**Keywords:** lung cancer, cancer stem cells, immune checkpoint inhibitors, EBUS/TBNA

## Abstract

Over the past decade, immune checkpoint inhibitors have revolutionized the treatment of non-small cell lung cancer (NSCLC). Unfortunately, not all patients benefit from PD-(L)1 blockade, yet, the PD-L1 tumor cell expression is the only approved biomarker, and other biomarkers have been investigated. In the present study, we analyzed the presence of immunomodulatory molecules: PD-L1, CD47, CD73, Fas, and FasL on mature tumor cells (MTCs) and cancer stem cells (CSCs) in lymph nodes (LNs) aspirates and refer it to the lymphocyte subpopulation in peripheral blood (PB). PB samples and LNs aspirates obtained during the endobronchial ultrasound-guided transbronchial needle aspiration (EBUS/TBNA) procedure of 20 patients at different stages of NSCLC. The cells were analyzed by multiparameter flow cytometry. We reported the higher frequency of MTCs and CSCs expressing the investigated immunomodulating molecules in metastatic LNs than in nonmetastatic. The expression of CD47 and PD-L1 was significantly higher on CSCs than on MTCs. Among the lymphocyte subpopulation in PB, we observed a higher frequency of PD-1+ CD8 T cells and Fas+ CD8 T cells in patients with confirmed metastases than in nonmetastatic. Next, we found that the percentage of FasL+ MTCs correlated with the frequency of Fas+ CD3 T cells in LNs aspirates and Fas+ CD8 T cells in PB. Finally, we found that patients with metastatic disease had a significantly higher FasL+/Fas+ MTCs ratio than patients with nonmetastatic disease. Both MTCs and CSCs express different immunomodulatory molecules on their surface. The frequency of FasL+ MTCs associates with altered distribution of Fas+ lymphocyte subpopulations in LNs and PB.

## 1. Introduction

Lung cancer remains the leading cause of cancer-related death worldwide [1,2]. Although small improvements have been made in the treatment of non-small cell lung cancer (NSCLC) over past decades, the efficacy of the new immunotherapy methods targeting immune checkpoints has been recently demonstrated in about 40% of patients [3,4]. More accurate biomarkers for individual therapy are needed.

Cancer stem cells (CSCs) are small numbers of cells that exist in the tumor microenvironment (TME) and hold stemness properties that sustain cancer progression, such as enhanced capacities for self-renewal cloning, growing, metastasizing, homing, and reproliferating [5]. Lung cancer TME is composed of a complex group of noncancer cells, such as stromal cells, tumor-associated macrophages, tumor-infiltrating lymphocytes, regulatory T cells, myeloid-derived suppressor cells, dendritic cells, NK cells, and natural killer T cells along with cancer cells: CSCs and mature tumor cells (MTCs). There are different accepted definitions for MTCs and CSCs regarding the surface markers they express. In the current state of knowledge, lung MTCs are described as CD45-/EpCAM+/CXCR4+ [6,7]. Lung CSCs are defined as CD45-/EpCAM+/CXCR4+/CD44+/CD133+/CD90+ [8,9,10,11]. It is considered that CSCs are responsible for recruiting noncancer cells to TME [12]. However, the interplay between CSCs and noncancer cells in TME, as well as interactions of CSCs with the immune system in the systemic circulation, is not fully understood. While there have been enormous advances in the understanding of the immunoediting process in the TME and its prognostic significance, less is known about changes in the regulatory mechanisms at the level of the LNs [13,14]. LNs are common sites of metastasis. Cancer cells that have metastasized to LNs may express immunosuppressive molecules and escape immune detection. Similar to primary TME, MTCs in metastatic LNs shape their interactions with the host immune system by controlling the infiltration and reactivity of immune cells [14]. At present, little is known about the frequency of immunomodulatory molecules on MTCs and CSCs in metastatic LNs in NSCLC patients.

As tumors develop, they acquire mechanisms to avoid attenuation by the immune system [15]. T cells play a key role in anticancer defense, but their population is modulated during the development of the disease [16]. The numerous suppressory and regulatory mechanisms inhibit the recognition of lung cancer antigens and are capable of blocking lymphocyte activation [17]. The strong suppressory pathway is the programmed death-1/programmed death ligand-1 (PD-1/PD-L1) pathway. Understanding these mechanisms can lead to the development of new strategies that provoke the immune system to recognize lung cancer as foreign. The success of checkpoint inhibitors creates interest in evaluating other antibodies that can be used to modulate T cell responses and/or enhance the ability of the immune system to destroy the tumor cells.

**Figure 1:**
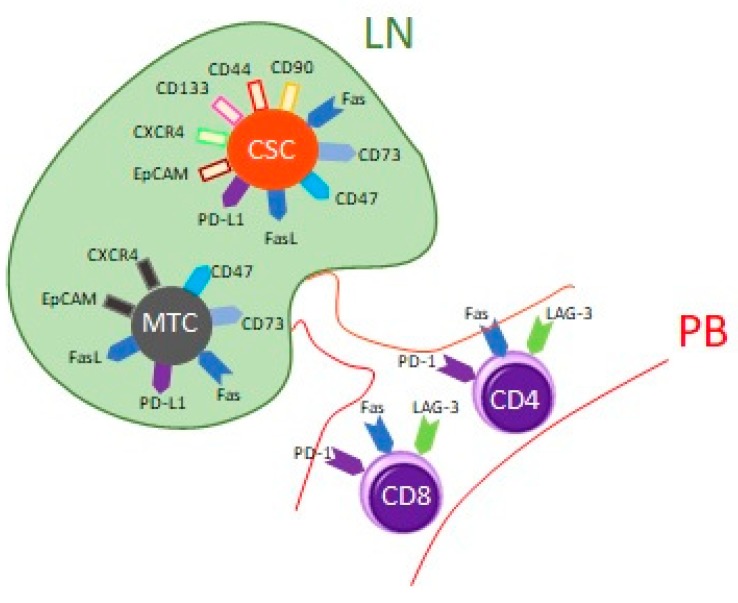
A study design. Mature tumor cells (MTC) were defined as CD45-/EpCAM+/CXCR4+; Cancer stem cells (CSCs) were defined as CD45-/EpCAM+/CXCR4+/CD133+/CD44+/CD90+. The presence of immunomodulatory molecules PD-L1, CD47, CD73, Fas, and FasL on MTC and CSCs in LNs were checked. Additionally, the presence of PD-1, Fas, and LAG3 on CD4 T cells and CD8 T cells in peripheral blood was evaluated.

In our previous study, we confirmed the presence of PD-L1+ CSCs in metastatic LNs in NSCLC patients that may suggest their immunosuppressive properties [18]. This study aimed to evaluate the presence of immunosuppressing molecules: PD-L1, CD47, CD73, Fas (CD95), and FasL (Fas Ligand) on CSCs and MTCs in LNs aspirates, and to evaluate the presence of PD-1 (programmed death receptor 1), Fas, and LAG3 (Lymphocyte-activation gene 3) on CD4 T cells and CD8 T cells in systemic circulation in NSCLC patients (Figure 1); based on the examination of the endobronchial ultrasound-guided transbronchial needle aspiration (EBUS/TBNA) technique and the peripheral blood (PB) from the same patient.

## 2. Results

### 2.1. Patients

The study group consisted of patients consecutively enrolled during diagnostic procedures for lung tumors. We qualified patients without any type of previous or recent anticancer therapy, clinical signs of infection, autoimmune diseases, or immunosuppressive treatment. Finally, only patients with histologically confirmed lung cancers were included in the study groups (*n* = 20). The current histological and TNM classification of lung cancer was used [19]. Each patient had provided written informed consent before EBUS/TBNA (the Medical University of Warsaw Ethics Committee: KB/230/2016). The group was divided according to the presence of LNs metastases confirmed histopathologically. The clinical characteristic of the lung cancer patients is summarized in Table A1. 

Molecular analysis was performed in 12 patients with adenocarcinoma or NOS (not otherwise specified). In five patients, an activated mutation of EGFR was confirmed, in two other patients, a rearrangement of ALK/EML4, and in two patient KRAS mutations were found.

### 2.2. MTCs and CSCs Frequencies in Peripheral Blood and LNs Aspirates

The gating strategy for MTCs and CSCs is presented in Appendix A Figure A2.

#### 2.2.1. LNs Aspirates

The analysis of LNs aspirates revealed the presence of MTCs in 17/20 samples. Higher percentage of MTCs was observed in metastatic LNs in the histological report than in nonmetastatic LNs (34.42% (10.0–65%) vs. 1.0% (0–5%), *p* < 0.05).

CSCs were found in 16/20 samples. Higher percentage of CSCs was observed in metastatic LNs than in nonmetastatic LNs (11.67% (4.9–23.4%) vs. 0.57% (0–3.0%), *p* < 0.05).

The percentage of MTCs was correlated with the percentage of CSCs (r = 0.8986, *p* < 0.05).

#### 2.2.2. Peripheral Blood Analysis

The identification of circulating MTCs and circulating CSCs was performed in 5 patients. The amount was too low to perform a proper analysis, and we decided not to conduct further analysis of MTCs and CSCs in PB in subsequent patients.

### 2.3. MTCs and CSCs Express Different Immunomodulatory Molecules on Their Surface

**Figure A1:**
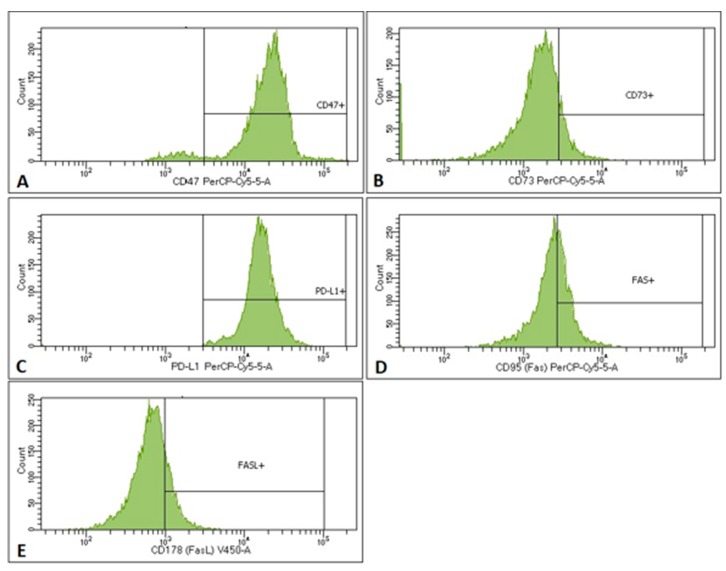
(**A**–**E**) Representative histograms used to assess the presence of immunomodulatory molecules on CSCs.

**Figure A2:**
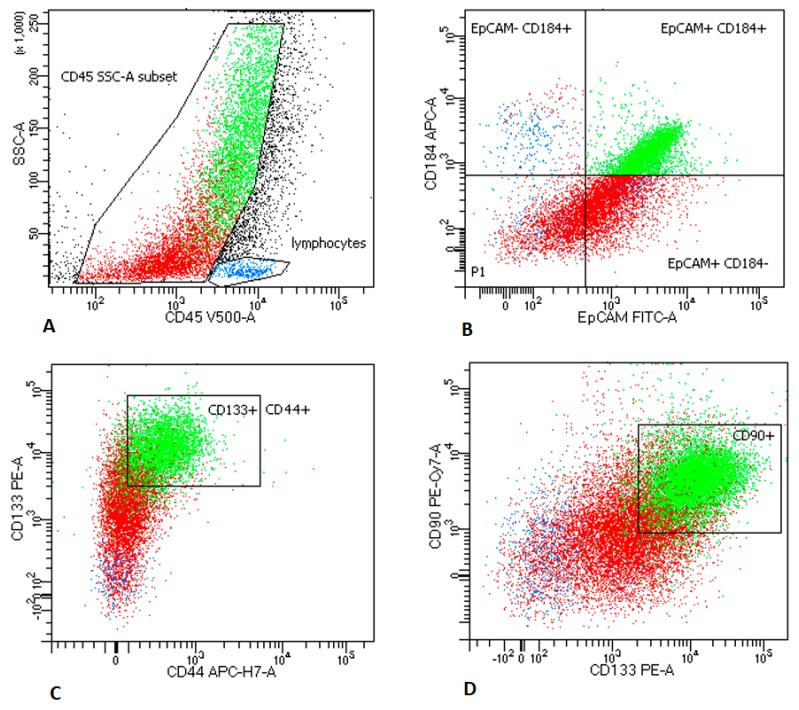
The gating strategy of cancer cells and CSCs in LNs aspirates. (**A**) The population of CD45- cells (nonlymphoid origin). (**B**) CD45-/CXCR4+/EpCAM+ gate; mature tumor cells (MTCs). (**C**) Among cancer cells, 32.5% cells express markers of stemness (CD44 and CD133). (**D**) Among CD45-/CD184+/CXCR4+/CD44+/CD133+ cells 35% of cells express additional marker of stemness CD90. CSCs represent 12.5% of all cells in LN aspirate.

We examined the expression of PD-L1, CD47, CD73, Fas, and FasL on MTC and lung CSCs in LNs aspirates. Representative histograms used to assess the presence of immunomodulatory molecules on CSCs are presented in Appendix A Figure A1. The frequencies of immunomodulatory molecules on MTCs were assessed in the same manner. 

The cell frequencies and the GMF (geometric mean fluorescence) intensity of PD-L1, CD47, CD73, Fas, and FasL on MTCs and CSCs are presented in Table A1.

**Figure 2:**
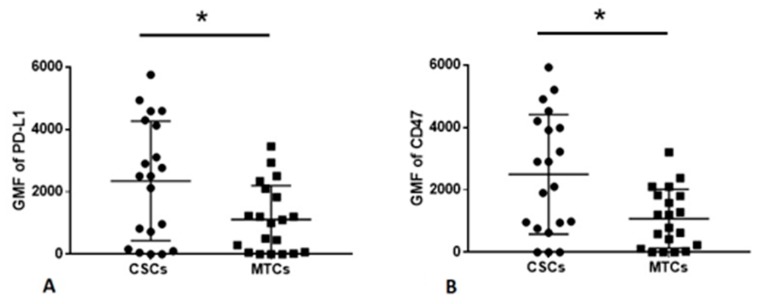
(**A**) The significant difference between the expression of PD-L1 on CSCs and MTCs, * *p* = 0.0481. (**B**) The significant difference between the expression of CD47 on CSCs and MTCs, * *p* = 0.0229. Data are expressed as median with interquartile range. * Differences between groups are significant in Mann–Whitney U-test.

Next, we compared the expression (GMF) of examined immunomodulatory molecules on CSCs and MTCs. We found that the PD-L1 and CD47 expression was increased on CSCs compared to MTCs (2246 vs. 1489; 2589 vs. 1346, respectively, *p* < 0.05) (Figure 2). We did not report any significant differences between the expression of Fas, FasL, and CD73 on CSCs and MTCs.

### 2.4. Frequencies of Lymphocyte Subsets in PB

**Figure A3:**
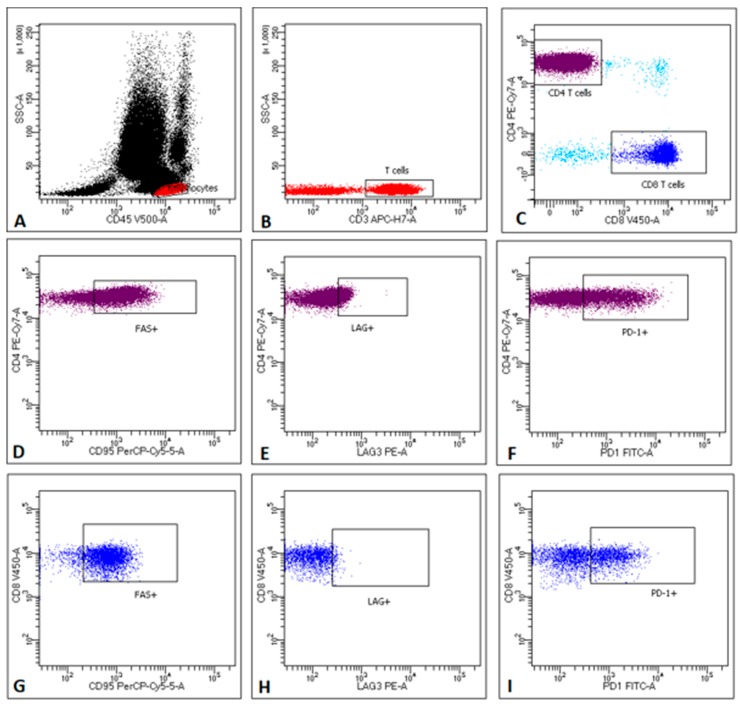
(**A**–**I**) The gating strategy for expression of Fas, PD-1, LAG3 on CD4+ T cells, and CD8+ T cells in PB.

The gating strategy for expression of Fas, PD-1, and LAG3 on CD4+ T cells and CD8+ T cells in PB is presented in Appendix A Figure A3.

**Figure 3:**
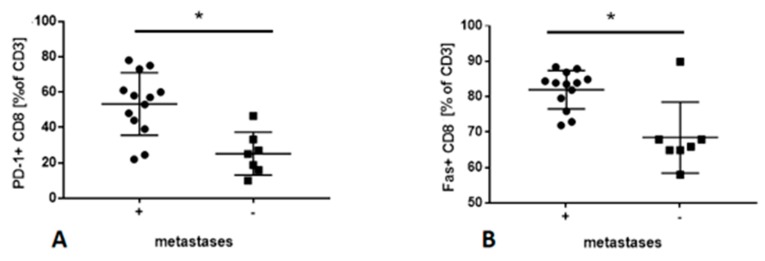
(**A**) The significant differences in the frequency of PD-1+ CD8 T cells in PB in patients with confirmed metastases in LNs and in patients without confirmed metastases in LNs, * *p* = 0.0034. (**B**) The significant differences in the frequency of Fas+ CD8 T cells in PB in patients with confirmed metastases in LNs and in patients without confirmed metastases in LNs, * *p* = 0.0078. Data are expressed as median with interquartile range. * Differences between groups are significant in Mann–Whitney U-test.

The frequency of CD4 and CD8 T cells in PB in patients with the metastatic and nonmetastatic disease was similar. However, patients with metastatic disease had a higher percentage of PD-1+ CD8+ T cells and Fas+ CD8 T cells than patients with nonmetastatic disease (51.86% (25.15% and 84.58% vs. 71.74% respectively *p* < 0.05) (Figure 3). There were no significant differences between other lymphocyte subpopulations in PB in patients with confirmed metastases in LNs and patients without confirmed metastases in LNs.

### 2.5. Fas/FasL Pathway

**Figure 4:**
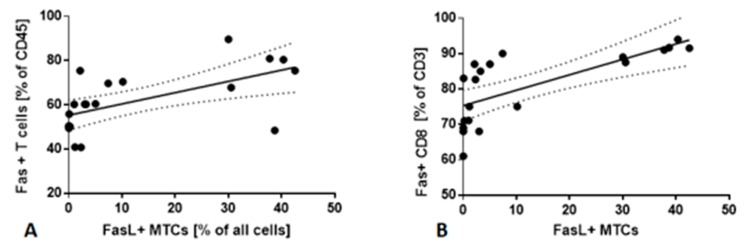
(**A**) The correlation between Fas+ CD3 T cells and FasL+ MTCs in LNs aspirates. (**B**) The correlation between Fas+ CD8 T cells in PB and FasL+ MTCs in LNs aspirates.

Flow cytometry analysis allowed assessing the presence of Fas and FasL on MTCs, CSCs, and T cells in LNs aspirates. Percentage of FasL+ MTCs correlated with Fas+ CD3 T cells in LNs aspirates (r = 0.8667; *p* < 0.05) (Figure 4A). We did not observe any significant correlations between FasL+ CSCs and Fas+ CD3 cells in LNs aspirates.

Next, we checked if there are significant dependencies between FasL+ MTCs, FasL+ CSCs in LNs, and Fas+ lymphocyte subsets in PB. We found that the percentage of FasL+ MTCs in LNs was correlated with the frequency of Fas+ CD8 T cells in PB (r = 0.7659; *p* < 0.05) (Figure 4B). No significant correlation between FasL+ CSCs in LNs and Fas+ T cells in PB was observed.

**Figure 5:**
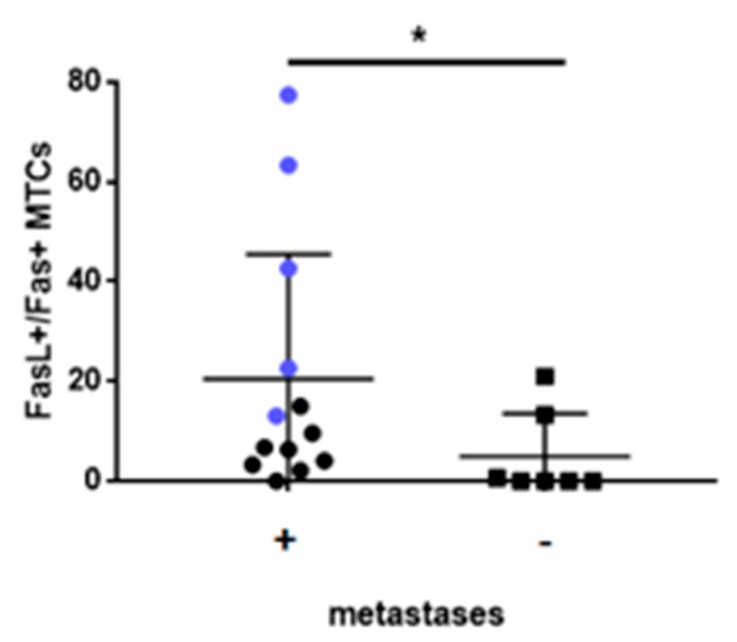
Significant differences between FasL+/Fas+ MTCs in LNs aspirates in patients with confirmed metastases in LNs and patients without confirmed metastases in LNs, * *p* = 0.0439. Data are expressed as median with interquartile range. * Differences between groups are significant in Mann–Whitney U-test. Blue dots represent patients with IV stage of the disease.

Finally, we observed that FasL+/Fas+ MTCs ratio in LNs differs between patients with metastatic and nonmetastatic disease. We found that patients with metastatic disease have a significantly higher FasL+/Fas+ MTCs ratio than patients with nonmetastatic disease (21.89 vs. 7.85; *p* < 0.05). Interestingly, the highest FasL+/Fas+ MTCs ratio was observed in patients with IV stage of the disease (Figure 5).

## 3. Discussion

We proved in our current study that the cellular composition of LNs could be measured during standard-of-care bronchoscopic assessment using flow cytometry. In contrast to standard immunohistochemistry, flow cytometry enables the simultaneous comprehensive analysis of many immunomodulatory markers in a single-cell population. The usefulness of this technique in the assessment of LNs in NSCLC patients has been confirmed by other authors [20,21]. For the first time, we detected CSCs and also MTCs, with the expression of molecules capable of modulation of the immune response, in LNs and PB of lung cancer patients. The interaction between CSCs and the immune system is not well understood and is currently of much interest. In our previous study, we confirmed the presence of PD-L1+ CSCs in LNs aspirates in NSCLC patients suggesting their immunogenic potential [18]. It encouraged us to investigate the presence of other immunomodulatory molecules on lung CSCs. The antibody panel we designed allows defining both CSC and MTC populations. We found that both CSCs and MTCs express all investigated immunomodulatory molecules: PD-L1, CD47, FasL, Fas, and CD73. The presence of these molecules was described on lung cancer cells and is adopted as a biomarker to immunotherapy [22,23,24,25]. To date, in the case of lung CSCs, only the presence of PD-L1 and CD47 has been presented [18,25]. 

We found that among all investigated immunomodulatory molecules, PD-L1 and CD47 have the highest expression (GMF) on CSCs and MTCs. The importance of PD-L1 for cancer immunology and treatment has become widely known. No defined clinical data are available in regard to CD47, but this molecule has generated significant interest to date [26]. In general, PD-L1 is a critical “do not find me” signal to the adaptive immune system [25], whereas CD47 is a critical “do not eat me” signal to the innate immune system, as well as a regulator of the adaptive immune response [27]. CD47 ablation stimulates macrophage phagocytosis and polarization and synergizes with PD-1 blockade [26]. Furthermore, anti-CD47 antibody or CD47 blockade treatments have been demonstrated to reduce tumor burden and increase patient survival in various tumor xenograft models [28]. 

Interestingly, we observed that GMF of both CD47 and PD-L1 was higher on CSCs than on MTCs. These results are in concordance with the study of Liu et al. [25], who reported the higher CD47 expression on CSCs than on tumor cells in lung cancer cell lines, using flow cytometry. One explanation might be that MTCs lose expression of some molecules during differentiation. The knockdown of CD47 suppressed certain stem-like properties of cancer cells, such as self-renewal and chemoresistance, suggesting that targeting CD47 could not only activate the phagocytosis of macrophages but also could be used to enhance treatment against CSCs [29]. Hence, CD47 expression may be another mechanism used by lung cancer cells, especially lung CSCs, to escape phagocytosis. These results may indicate CD47 as a therapeutic target in lung cancer, as well as a therapeutic target in lung CSCs.

We noticed the presence of CD73 on the CSCs and MTCs population. The expression of CD73 in the tumor TME has been described in various types of cancer and is at least partly driven by hypoxia and activation of hypoxia-inducible (HIF) transcription factors [30]. Hypoxia drives expression of the well-defined transcription factor HIF1α, which promotes the expression of ectoenzymes CD39 and CD73 on tumor cells, stromal cells, and tumor-infiltrating immunosuppressive cell subsets, such as regulatory T cells (Tregs) and myeloid-derived suppressor cells (MDSCs) [31]. CD39 catalyzes the conversion of ATP and ADP into AMP, while CD73 catalyzes the irreversible conversion of AMP into adenosine [31]. CD73-derived adenosine accumulates in the TME and exerts multiple immunosuppressive actions to dampen antitumor immunity, leading to worse clinical outcomes [30]. Furthermore, CD73 has been shown to be biomarkers of patient outcomes in several tumor types, including NSCLC [23]. It has been described that CD73 promotes the expression of stemness and epithelial-mesenchymal transition (EMT), implying a regulation of CSCs function in ovarian cancer [32]. Anti-CD73 antibodies were shown to reduce tumor growth and metastasis through the activation of NK and T cell responses [31]. Recently, CD73 has been an extensively investigated target in anticancer therapy and shows synergy with anti-PD-1/PD-L1 agents [33].

The Fas/FasL system is involved in programmed cell death. Fas bearing cells are susceptible to apoptosis induced by connection with Fas ligand (FasL). It was observed that tumor cells that express FasL induce apoptosis of lymphocytes expressing Fas. On the other hand, cancer cells are resistant to apoptosis, and the Fas/FasL system is impaired. In our study, we have found that lung cancer cells express both Fas and FasL, which is in concordance with a study by Li et al. [34]. The vast majority of reports demonstrate that the expression of both Fas and especially of FasL acts as a negative prognostic marker for many cancers [24]. In our group, the reduced Fas expression on MTCs was found more frequently in the advanced stage than in the earlier stages. It also appeared to be significantly associated with higher nodal status.

What is more, we found that a FasL/Fas ratio was significantly higher in patients with confirmed metastases in LNs than in patients without metastatic disease. These associations suggest that defects in the apoptotic pathway represent a significant element in the progression of the NSCLCs. Altogether, these data support a role for the loss of Fas-mediated apoptosis during tumorigenesis and tumor progression. 

The best to our knowledge, we report the presence of Fas and FasL on lung CSCs for the first time. The concept that Fas can be a tumor promoter has now gained wide acceptance, supported by several reports describing the marked activities of Fas in tumor growth and spread [24]. Moreover, and related to this, Fas is capable of inducing the EMT process in gastrointestinal and breast cancer [35,36]. In both studies, stimulation of Fas on cancer cells induced a conversion from non-CSCs to CSCs, in consequence, increasing the frequency of CSCs. Unfortunately, the molecular mechanisms underlying the switch between these different signaling pathways remain enigmatic.

FasL has a range of tumor-promoting activities, some of which are indirect, such as the suppression of the immune response in the cancer microenvironment, by killing Fas positive immune cells [19]. We examined the presence of Fas on CD3 T cells in LNs and the presence of Fas on CD4 and CD8 T cells in PB. We found that Fas+ CD3 T cells were significantly correlated with FasL+ MTCs in LNs aspirates. There was a higher frequency of Fas+ CD8 T cells in PB in NSCLC patients with confirmed metastases than in patients without metastases. An elevated proportion of PB lymphocytes with Fas expression was previously reported in patients with lung cancer and COPD [37,38]. Other authors have reported the higher percentage of Fas+ CD8 T in malignant pleural effusion (what may represent the tumor milieu), but not PB in lung cancer patients [39]. It should be noted that the proportion of Fas+ lymphocytes is usually correlated with the intensity of tobacco exposure that leads to chronic inflammation [37,38]. Here, we did not observe any significant correlation between the frequency of Fas+ lymphocytes and pack-years smoked. 

In our previous study, we found some correlation between suppressory immune cells in LNs and PD-L1+ CSC [40]. Here, we did not observe any significant dependencies between PD-L1+ CSCs, CD47+ CSCs, CD73+ CSCs population, and immunophenotype of lymphocyte in PB. In all, it seems that all examined immunomodulatory molecules may be involved in different pathways leading to tumor escape from immune surveillance, and each of them requires further investigation. 

Finally, we found that among 7 samples classified as nonmetastatic in the histopathological examination, MTCs were found in 4 samples and CSCs in 3 samples in multiparameter flow cytometry assay; among these four patients, three were at the IIB stage of NSCLC and one at the IB stage of NSCLC. Immunohistochemistry remains the ‘gold standard’ in assessing LNs involvement [41]. Research by other authors also showed the feasibility of EBUS/TBNA samples of LNs for flow cytometric analysis of MTCs [42]. Moreover, a retrospective study performed by Gwozdz et al. demonstrated that the presence of occult micrometastases in the mediastinal LNs was associated with reduced survival in I and II stage NSCLC patients due to tumor recurrence [43]. Their and our study demonstrated the usefulness of the EpCAM marker for the detection of early LNs cancer invasion. 

We are aware that the investigated group is low. The sample size did not allow us to perform a reliable comparison between the histological subtypes of NSCLC and between patients with confirmed genetic alterations mutations. Another weakness of our study was the lack of follow-up and comparison of our findings to the course of the disease. Thus our results indicate and support the direction for further investigations. 

## 4. Materials and Methods 

We obtained 2 ml of peripheral blood (PB) and placed it in tubes containing K2EDTA and processed for flow cytometry.

LNs group 4, 7, 10, and 11 aspirates were obtained during routine EBUS/TBNA procedure of lung cancer diagnosis. After diagnostic aspiration, the additional sample was taken for flow cytometry analysis. About 1 ml of LNs aspirate was diluted in 0.9% NaCl, collected in tubes containing K2EDTA, and processed for flow cytometry.

To identify MTCs flow cytometry and staining using monoclonal antibodies targeting the cell-surface expression of CD45 (V450), EpCAM (FITC) and CXCR4 (APC) were used (BD, USA). MTCs were defined as CD45-/EpCAM+/CXCR4+. CSCs population was determined by a panel of monoclonal antibodies: EpCAM (FITC), CD133 (PE), CD90 (PE-Cy7), CXCR4 (APC), CD44 (APC-H7), and CD45 (V500). CSCs were defined as: CD45-/CXCR4+/EpCAM+/CD133+/CD44+/CD90+. 

Additionally, anti-PD-L1 (PerCP-Cy5.5), anti-CD47 (PerCP-Cy5.5), anti-CD73 (PerCP-Cy5.5), anti Fas (PerCP-Cy5.5), and FasL (V450) antibodies were applied to assess the presence of these molecules on MTC and CSCs in LNs aspirates.

The proportion of CD4+ or CD8+ subpopulations in PB and LNs aspirates were determined by a panel of monoclonal antibodies: anti CD45 (V500), anti CD3 (APC-H7), anti CD8 (V450), and anti CD4 (PE-Cy7). 

Additionally, anti-PD-1 (FITC), anti-Fas (PerCP-Cy5.5), and anti-Lag3 (PE) antibodies were applied to assess the presence of these molecules on CD4 and CD8 T cells in PB. Briefly, preparation for flow cytometry was as follows: to each cytometric tube, 100 μL of LNs aspirate or PB and 4 μL of specific monoclonal antibodies were added. After 15 min of incubation in the dark, at room temperature, erythrocytes were lysed with lysing solution for 10 min and washed with 2% newborn calf serum in physiological buffer solutions (PBS). The cells were subsequently fixed in PBS. The samples were processed by the FACS Canto II flow cytometer (BD, USA). Geometric mean fluorescence (GMF) intensity of PD-L1, CD47, CD73, Fas, and FasL on MTCs and CSCs was measured. The analysis was performed using BD FACSDiva™ Software (BD, USA)

For the statistical analysis, the Mann–Whitney U-test was performed to: compare the differences between patients with confirmed metastases in LNs and patients without metastases, and compare the differences between the expression of immunomodulatory molecules on CSCs and MTCs. Correlation analyses were performed by calculating the Pearson r coefficient. Differences were considered statistically significant when *p* < 0.05. All analyses were performed using Prism (Version 5, GraphPad Software, La Jolla, CA, USA). 

## 5. Conclusions

In conclusion, we reported the higher frequency of MTCs and CSCs expressing investigated immunomodulating molecules in metastatic LNs than in nonmetastatic. Moreover, we found some significant dependencies between FasL+ MTCs and Fas+ lymphocytes in LNs aspirates and PB. Additionally, we confirmed the utility of flow cytometric analysis of EBUS/TBNA samples in assessing the presence of metastases and cellular composition of LNs in NSCLC patients. 

## Appendix A

**Table A1:** The characteristics of the investigated group, the cell frequencies, and the GMF intensity of PD-L1, CD47, CD73, Fas, and FasL on CSCs. Data are expressed as median values (p25–p75). m, male; f, female; ADC, adenocarcinoma; SQCLC, squamous cell carcinoma; ASC, adenosquamous carcinoma; LCNEC, large cell neuroendocrine carcinoma; NOS, not otherwise specified.

Case	Investigated LN	Sex/age	Histological Subtype	EGFR/ALK/KRAS	Stage of Cancer	CSCs % of All Cells/Geomean				
						PD-L1+	CD47+	CD73+	Fas+	FasL+
**LNs with confirmed metastases**										
**1.**	**11**	**m/65**	**ADC**	-/+/-	IIIA	7.58/4194	8.40/4200	1.70/1264	2.12/849	2.60/1600
2.	4	f/74	LCNEC		IIIA	3.83/2502	3.50/2246	0.42/1201	0.89/519	1.20/943
3.	4	m/62	NOS	+/-/-	IIIA	2.20/565	1.99/687	0.02/128	0.98/34	1.62/113
4.	7	m/69	ADC	-/-/-	IIIC	6.70/2650	3.00/2831	2.60/762	3.20/480	3.74/745
5.	4	m/64	ADC	-/-/+	IV	15.50/4133	14.50/5923	5.33/3155	0.69/209	5.90/2351
6.	7	f/67	ADC	+/-/-	IIIB	10.20/2091	9.50/934	2.41/1928	5.50/850	6.80/1900
7.	4	m/67	SQCLC		IIIA	5.90/2154	4.80/1900	3.54/637	1.18/134	5.20/1987
8.	7	f/71	ADC	+/-/-	IV	9.84/4123	7.86/3724	5.54/2753	1.80/235	6.47/3291
9.	7	m/58	ADC	+/-/-	IV	6.60/3109	4.90/2523	2.60/823	1.10/198	4.08/765
10.	11	m/64	SQCLC		IIIB	2.87/1123	2.54/987	0.98/346	0.40/90	1.10/680
11.	4	m/72	ADC	-/-/+	IV	12.20/4581	10.89/5201	6.70/1932	2.20/847	5.44/830
12.	4	M/66	SQCLC		IV	10.20/2454	8.74/3954	4.87/3645	1.90/658	2.15/745
13.	7	F/67	ADC	-/+/-	IIIC	9.40/3654	6.53/4380	2.98/1245	2.10/487	3.65/2300
**LNs without confirmed metastases**										
1.	4	m/66	SQCLC		IB	0.50/723	0.48/748	0.27/532	0.20/75	0.30/180
2.	4	m/69	ASC		IB	0.09/222	0.08/123	0.02/143	0.07/60	0.02/90
3.	11	f/69	ASC		IIB	0.01/250	0.05/99	0.00/160	0.03/50	0.15/164
4.	4	f/60	NOS		IIB	0.00/0	0.00/0	0.00/0	0.00/0	0.00/0
5.	11	f/64	SQCLC		IIIA	0.00/0	0.00/0	0.00/0	0.00/0	0.00/0
6.	11	f/68	ADC	+/-/-	IIIA	2.50/524	2.10/490	1.89/410	1.96/75	2.30/179
7.	4	f/59	ADC	-/-/-	IIB	0.01/50	0.00/0	0.01/0	0.00/0	0.01/25

## References

[1] Ferlay J., Ervik M., Lam F., Colombet M., Mery L., Piñeros M., Znaor A., Soerjomataram I., Bray F. (2018). GLOBOCAN 2018 v1.0, Cancer Incidence and Mortality Worldwide: IARC Cancer Base No. 15. https://gco.iarc.fr.

[2] Rahib L., Smith B.D., Aizenberg R., Rosenzweig A.B., Fleshman J.M., Matrisian L.M. (2014). Projecting cancer incidence and deaths to 2030: The unexpected burden of thyroid, liver, and pancreas cancers in the United States. Cancer Res..

[3] Costantini A., Grynovska M., Lucibello F., Moisés J., Pagès F., Tsao M.S., Shepherd F.A., Bouchaab H., Garassino M., Aerts J.G.J.V. (2018). Immunotherapy: A new standard of care in thoracic malignancies? A summary of the European Respiratory Society research seminar of the Thoracic Oncology Assembly. Eur. Respir. J..

[4] Dudnik E., Moskovitz M., Daher S., Shamai S., Hanovich E., Grubstein A., Shochat T., Wollner M., Bar J., Merimsky O. (2017). Effectiveness and safety of nivolumab in advanced non-small cell lung cancer: The real-life data. Lung Cancer.

[5] Chen W., Dong J., Haiech J., Kilhoffer M.C., Zeniou M. (2016). Cancer Stem Cell Quiescence and Plasticity as Major Challenges in Cancer Therapy. Stem Cells Int..

[6] Nicolazzo C., Raimondi C., Mancini M., Caponnetto S., Gradilone A., Gandini O., Mastromartino M., del Bene G., Prete A., Longo F. (2016). Monitoring PD-L1 positive circulating tumor cells in non-small cell lung cancer patients treated with the PD-1 inhibitor Nivolumab. Sci. Rep..

[7] Xie S., Zeng W., Fan G., Huang J., Kang G., Geng Q., Cheng B., Wang W., Dong P. (2014). Effect of CXCL12/CXCR4 on increasing the metastatic potential of non-small cell lung cancer in vitro is inhibited through the downregulation of CXCR4 chemokine receptor expression. Oncol. Lett..

[8] Skirecki T., Hoser G., Kawiak J., Dziedzic D., Domagała-Kulawik J. (2014). Flow cytometric analysis of CD133- and EpCAM-positive cells in the peripheral blood of patients with lung cancer. Arch. Immunol. Ther. Exp..

[9] Barr M.P., Gray S.G., Hoffmann A.C., Hilger R.A., Thomale J., O’Flaherty J.D., Fennell D.A., Richard D., O’Leary J.J., O’Byrne K.J. (2013). Generation and characterisation of cisplatin-resistant non-small cell lung cancer cell lines displaying a stem-like signature. PLoS ONE.

[10] Zhang W.C., Shyh-Chang N., Yang H., Rai A., Umashankar S., Ma S., Soh B.S., Sun L.L., Tai B.C., Nga M.E. (2012). Glycine decarboxylase acticity drives non-small cell lung cancer tumorinitiating cells and tumorigenesis. Cell.

[11] Wang P., Gao Q., Suo Z., Munthe E., Solberg S., Ma L., Wang M., Westerdaal N.A.C., Kvalheim G., Gaudernack G. (2013). Identification and characterization of cells with cancer stem cell properties in human primary lung cancer cell lines. PLoS ONE.

[12] Badrinath N., Yoo S.Y. (2019). Recent Advances in Cancer Stem Cell-Targeted Immunotherapy. Cancers.

[13] Ogino S., Galon J., Fuchs C.S., Dranoff G. (2011). Cancer immunology—Analysis of host and tumor factors for personalized medicine. Nat. Rev. Clin. Oncol..

[14] Jones D., Pereira E.R., Padera T.P. (2018). Growth and Immune Evasion of Lymph Node Metastasis. Front. Oncol..

[15] Schreiber R.D., Old L.J., Smyth M.J. (2011). Cancer immunoediting: Integrating immunity’s roles in cancer suppression and promotion. Science.

[16] Aerts J.G., Hegmans J.P. (2013). Tumor-specific cytotoxic T cells are crucial for efficacy of immunomodulatory antibodies in patients with lung cancer. Cancer Res..

[17] Domagala-Kulawik J. (2015). The role of the immune system in non-small cell lung carcinoma and potential for therapeutic intervention. Transl. Lung Cancer Res..

[18] Raniszewska A., Polubiec-Kownacka M., Rutkowska E., Domagała-Kulawik J. (2019). PD-L1 expression on lung cancer stem cells in metastatic lymph nodes aspirates. Stem Cell Rev..

[19] Detterbeck F.C., Boffa D.J., Kim A.W., Tanoue L.T. (2017). The eighth edition lung cancer stage classification. Chest.

[20] Murthy V., Katzman D.P., Tsay J.J., Bessich J.L., Michaud G.C., Rafeq S., Minehart J., Mangalick K., de Lafaille M.A.C., Goparaju C. (2019). Tumor-draining lymph nodes demonstrate a suppressive immunophenotype in patients with non-small cell lung cancer assessed by endobronchial ultrasound-guided transbronchial needle aspiration: A pilot study. Lung Cancer.

[21] van de Ven R., Niemeijer A.N., Stam A.G.M., Hashemi S.M., Slockers C.G., Daniels J.M., Thunnissen E., Smit E.F., de Gruijl T.D., de Langen A.J. (2017). High PD-1 expression on regulatory and effector T-cells in lung cancer draining lymph nodes. ERJ Open Res..

[22] Lian S., Xi R., Ye Y., Lu Y., Cheng Y., Xie X., Li S., Jia L. (2019). Dual blockage of both PD-L1 and CD47 enhances immunotherapy against circulating tumor cells. Sci. Rep..

[23] Inoue Y., Yoshimura K., Kurabe N., Kahyo T., Kawase A., Tanahashi M., Ogawa H., Inui N., Funai K., Shinmura K. (2017). Prognostic impact of CD73 and A2A adenosine receptor expression in non-small-cell lung cancer. Oncotarget.

[24] Peter M.E., Hadji A., Murmann A.E., Brockway S., Putzbach W., Pattanayak A., Ceppi P. (2015). The role of CD95 and CD95 ligand in cancer. Cell Death Differ..

[25] Liu L., Zhang L., Yang L., Li H., Li R., Yu J., Yang L., Wei F., Yan C., Sun Q. (2017). Anti-CD47 Antibody As a Targeted Therapeutic Agent for Human Lung Cancer and Cancer Stem Cells. Front. Immunol..

[26] Weiskopf K. (2017). Cancer immunotherapy targeting the CD47/SIRPα axis. Eur. J. Cancer.

[27] Jaiswal S., Jamieson C.H., Pang W.W., Park C.Y., Chao M.P., Majeti R., Traver D., van Rooijen N., Weissman I.L. (2009). CD47 is upregulated on circulating hematopoietic stem cells and leukemia cells to avoid phagocytosis. Cell.

[28] Horrigan S.K. (2017). Reproducibility Project: Cancer B. Replication Study: The CD47-signal regulatory protein alpha (SIRPa) interaction is a therapeutic target for human solid tumors. eLife.

[29] Wang J.H., Huang S.T., Zhang L., Liu Z.G., Liang R.X., Jiang S.W., Jiang Y.N., Yu X.J., Jiang Y.C., Li X.Z. (2019). Combined prognostic value of the cancer stem cell markers CD47 and CD133 in esophageal squamous cell carcinoma. Cancer Med..

[30] Allard B., Longhi M.S., Robson S.C., Stagg J. (2017). The ectonucleotidases CD39 and CD73: Novel checkpoint inhibitor targets. Immunol. Rev..

[31] Sek K., Mølck C., Stewart G.D., Kats L., Darcy P.K., Beavis P.A. (2018). Targeting Adenosine Receptor Signaling in Cancer Immunotherapy. Int. J. Mol. Sci..

[32] Lupia M., Angiolini F., Bertalot G., Freddi S., Sachsenmeier K.F., Chisci E., Kutryb-Zajac B., Confalonieri S., Smolenski R.T., Giovannoni R. (2018). CD73 Regulates Stemness and Epithelial-Mesenchymal Transition in Ovarian Cancer-Initiating Cells. Stem Cell Rep..

[33] Mazzarella L., Duso B.A., Trapani D., Belli C., D’Amico P., Ferraro E., Viale G., Curigliano G. (2019). The evolving landscape of ‘next-generation’ immune checkpoint Inhibitor: A Review. Eur. J. Cancer.

[34] Li Y., Xu K.P., Jiang D., Zhao J., Ge J.F., Zheng S.Y. (2015). Relationship of Fas, FasL, p53 and bcl-2 expression in human non-small cell lung carcinomas. Int. J. Clin. Exp. Pathol..

[35] Zheng H., Li W., Wang Y., Xie T., Cai Y., Wang Z., Jiang B. (2014). miR-23a inhibits E-cadherin expression and is regulated by AP-1 and NFAT4 complex during Fas-induced EMT in gastrointestinal cancer. Carcinogenesis.

[36] Ceppi P., Hadji A., Kohlhapp F.J., Pattanayak A., Hau A., Liu X., Liu H., Murmann A.E., Peter M.E. (2014). CD95 and CD95L promote and protect cancer stem cells. Nat. Commun..

[37] Domagala-Kulawik J., Hoser G., Dabrowska M., Chazan R. (2007). Increased proportion of Fas positive CD8+ cells in peripheral blood of patients with COPD. Respir. Med..

[38] Hoser G., Wasilewska D., Domagala-Kulawik J. (2004). Expression of Fas receptor on peripheral blood lymphocytes from patients with non-small cell lung cancer. Folia Histochem. Cytobiol..

[39] Prado-Garcia H., Romero-Garcia S., Aguilar-Cazares D., Meneses-Flores M., Lopez-Gonzalez J.S. (2012). Tumor-induced CD8+ T-cell dysfunction in lung cancer patients. Clin. Dev. Immunol..

[40] Raniszewska A., Vroman H., Dumoulin D., Domagala-Kulawik J., Aerts J.G.J.V. (2019). Immunomodulating properties of PD-L1 positive cancer stem cells in metastatic lymph nodes. Eur. Respir. J..

[41] Planchard D., Popat S., Kerr K., Novello S., Smit E.F., Faivre-Finn C., Mok T.S., Reck M., van Schil P.E., Hellmann M.D. (2019). Metastatic non-small cell lung cancer: ESMO Clinical Practice Guidelines for diagnosis, treatment and follow-up. Ann. Oncol..

[42] Bugalho A., Martins C., Dias S.S., Nunes G., Silva Z., Correia M., Gomes M.J.M., Videira P.A. (2013). Cytokeratin 19, carcinoembryonic antigen, and epithelial cell adhesion molecule detect lung cancer lymph node metastasis in endobronchial ultrasound-guided transbronchial aspiration samples. Clin. Lung Cancer.

[43] Gwóźdź P., Pasieka-Lis M., Kołodziej K. (2018). Prognosis of patients with stages I and II non-small cell lung Cancer with nodal micrometastases. Ann. Thorac. Surg..

